# Medicine use practices in management of symptoms of acute upper respiratory tract infections in children (≤12 years) in Kampala city, Uganda

**DOI:** 10.1186/s12889-017-4770-1

**Published:** 2017-09-21

**Authors:** Moses Ocan, Mary Aono, Clare Bukirwa, Emmanuel Luyinda, Cathy Ochwo, Elastus Nsambu, Stella Namugonza, Joseph Makoba, Enock Kandaruku, Hannington Muyende, Aida Nakawunde

**Affiliations:** 1 Department of Medicine, College of Health Sciences, Makerere University, P.O. Box 7072, Kampala, Uganda; 2 Department of Pharmacology & Therapeutics, College of Health Sciences, Makerere University, P.O. Box 7072, Kampala, Uganda; 3 Department of Pharmacy, College of Health Sciences, Makerere University, P. O. Box, 7072 Kampala, Uganda

**Keywords:** Upper respiratory tract infections, Self-medication, Antimicrobial agents, Kampala

## Abstract

**Background:**

Medicines are commonly accessed and used for management of illness in children without a prescription. This potentially increases the risk of unwanted treatment outcomes. We investigated medicine use practices in management of symptoms of acute upper respiratory tract infections among children (≤12 years) in households in Nakawa division, Kampala city.

**Methods:**

This was a cross-sectional study conducted among 390 randomly selected children. Data on use of medicines in children (≤12 years) during recent episode of acute upper respiratory tract infection was collected from their care takers using an interviewer administered questionnaire. A recall period of two weeks (14 days) was used in during data collection.

**Results:**

The prevalence of giving children non-prescription antimicrobial medicines was 44.8% (38.3-52.2). The most common disease symptoms that the children reportedly had included flu, 84.9% (331/390), cough, 83.1% (324/390), and undefined fever, 69.7% (272/390). Medicines commonly given to children included, paracetamol 53.1% (207/390), Coartem 29.7% (116/390), cough linctus 20.8% (81/390), amoxicillin 18.9% (74/390), Co-trimoxazole 18.5% (72/390), and diphenhydramine 15.4% (60/390). The major sources of medicines given to the children was hospital/clinic, 57.26% (223/390). Most of the children, 81% were given more than one medicine at a time. The majority, 62.3% (243/390) of the care takers who gave the children medicine during the recent illness were not aware of any medicine (s) that should not be given to children. The predictors of non-prescription use of antimicrobial medicines in managing symptoms of acute upper respiratory tract infections in children included, medicines obtained from drug shop (PR: 1.45, CI: 1.14-1.85), medicines at home (PR: 1.8, CI: 0.83-1.198) and type of medicine (antimalarial) (PR: 2.8, CI: 1.17-6.68).

**Conclusion:**

Children are commonly given multiple medicines during episodes of acute upper respiratory tract infections with most antimicrobial agents accessed and used without a prescription in Kampala city, Uganda.

## Background

The use of medicines to manage illnesses in children is a common practice in most parts of the world [1]. Disease symptoms such as flu, sinusitis, laryngitis, cough, fever, rhinorrhea, sneezing, sore throat and myalgia are among the major drivers of medicine use in children [2]. In Uganda, acute respiratory tract infections are among the leading causes of morbidity and mortality in children [3]. Children are the main users of healthcare services in both private and public health sectors in most developing countries [1].

The medicines used for illness management in children are commonly obtained without a prescription which potentially increases the risk of inappropriate use [4]. World Health Organization (WHO) estimates that more than half of all medicines globally are used without a prescription [5]. A study done in northern Uganda showed that majority, 75.7% of adult (≥18 years) community members access and use antimicrobial agents without a prescription [6].

Information on medicine safety in children is mostly limited due to lack of clinical trials conducted in children globally [7, 8]. Therefore use of medicines in children is commonly extrapolated and in some cases adopted from how they are used in adults [9]. Observations of treatment outcomes in adults thus forms the basis for selecting the medicines used in children. However, such decisions are in most cases made by the caretakers of children who lack biomedical knowledge of the medicines or the disease process. This could increase the risk of mistreatment and the associated adverse effects [10]. For example, during 2004 -to- 2005, an estimated 1519 children <2 years of age were treated in emergency departments of hospitals across the USA for adverse events associated with cough and cold medicines [11].

A third, 30.9% of the population in Uganda fall in the age group 0–8 years and a further 21.3% are in the age group 6–12 years. In the recent national census, 55% of the population is below the age of 18 years (children as per constitution) in Uganda [12]. In addition, this age group has high mortality rate in Uganda as per the most recent census report of 2014. For example, in Uganda under five mortality rate is 80 deaths per 1000 live births [12]. There is thus a high likelihood of inappropriate medicine use among this population in Uganda. However, in most developing countries like Uganda, there is limited information on medicine use in managing illnesses in children, associated effects and factors influencing the practice. This study was thus intended to investigate the prevalence, practices and factors associated with medicine use in management of symptoms of acute upper respiratory tract infections in children (≤12 years) in households of Nakawa division, Kampala city.

## Methods

### Study design and setting

This was a cross-sectional study done in Kampala city. Data was collected in Nakawa division a residential, commercial and industrial area in Kampala city. It accommodates over 2000 families, with average of two (2) adults and five (5) children per family [12]. Most families stay in temporary make shift shelters. In this suburb, majority of the population earn a living from small scale businesses.

Nakawa division which is one of the five administrative divisions of Kampala city, was randomly selected for this study using simple random sampling method. Briefly, the names of the five administrative divisions of Kampala city (Kawempe division, Makindye division, Rubaga division, Nakawa dividion and Central division) were each written in small pieces of paper which were then folded and placed in a basket. The basket was shaken and one piece of paper picked. The division whose name was in the paper (Nakawa) was used for data collection in this study.

### Study population and sample size

The study was done in children (≤12 years) of age in Nakawa division. We included all children (≤12 years) in households who had an episode of acute upper respiratory tract infection in the last two (14) weeks prior to the date of data collection. However, the children (≤12 years) who were on antimicrobial agents for chronic treatment were excluded from the study.

The primary outcome of this study was, the prevalence of use of antimicrobial agents without a prescription in management of symptoms of acute upper respiratory tract infections in children in Nakawa division, Kamapala city. The study sample size was calculated using the formula by Kish and Leslie [13].

$$ n=\frac{z_{\alpha /2}^2p\left(1-p\right)}{d^2} $$

Where; n = Sample size; Z_α_ = is the abscissa of the normal distribution curve and its value, 1.96 for a 95% confidence level is found in statistical tables which contain the area under the normal curve. d = desired level of precision (5%), p = is the estimated proportion of an attribute (prevalence of non-prescription use of medicines in childhood illnesses; *p* = 50%). After substitution, a sample size of 384 children was obtained for the study to detect the main outcome at 95% confidence level.

### Sampling procedure

Systematic random sampling procedure was used. The study team first identified and visited the house of a local council one area leader in Nakawa division for permission to conduct the study. From the residence of the local area leader, the study team then separated and moved in three separate directions. Every third house in the direction taken by either of the study teams was sampled for the study. If the selected household did not have any child below the age of ≤12 years who had a recent episode of acute upper respiratory tract infection, it was replaced by the next nearest household. Households that had more than one child that met the inclusion criteria (≤12 years of age), data on medicine use was collected for only one randomly selected child. This was done by writing the names of all the eligible children on small pieces of paper, folded and put in a basket. One piece of paper was then picked and data on medicine use was collected for the child whose name appeared on the paper.

### Data collection procedure

The data collection tool was pre-tested using ten (10) respondents (childcare takers) in households in Kawempe one of the five administrative divisions of Kampala city. The results of the pre-test were used to adjust the tool. The questions of the data collection tool were initially derived from literature review especially from articles by Morgan et al. [14] and Ocan et al. [6] and then further adjusted using the outcome of the pre-test. Data was collected from the selected respondents using a pre-tested structured interviewer administered questionnaire. The interviewers were trained on the tool and participated in pre-testing of the tool. Ten (10) third year medical students of Makerere University College of Health Sciences were first trained using the data collection tool prior to field data collection.

The data collection tool had the following questions: i) demographic information, ii) has the child been ill in the last two weeks, iii) what symptoms did the child have, iv) what disease do you think the child was suffering from, v) during this illness, did the child receive any treatment, vi) did the child take medicine without a prescription during the recent illness, vii) who recommended the treatment, viii) which medicine(s) was the child given, ix) what was the source of the medicine, x) did the child complete taking all the medicines obtained, xi) what did you do with the medicines which remained, xii) was the child given more than one medicine at a time, xiii) did the child improve, xiv) if no, what did you do for the child’s illness, xv) are you aware of the risks of taking medicines without a prescription in children, xvi) what are some of these risks, xvii) which medicines are contraindicated in children, xviii) In case of future illness would you treat the child without consulting a healthcare professional, xix) would you recommend self-medication for other children in the neighborhood.

Where possible, the childcare takers were at each time requested to provide samples of any medicines that they have been giving to the children if they still had them or the packaging material of the medicines that were given to the children. These were assessed to help corroborate self-reported information on medicines that were given to children during the illness.

### Data management and analysis

Field data collection was done from May-to-June, 2015. At the end of each data collection day, all the filled questionnaires were reviewed for completeness and consistency by the principal investigators (MO and AO). The errors in different forms were identified, shared and missing information re-collected from the same household. Data from the questionnaires were double entered in Epi-data 3.1 software. The two files were merged in STATA 12.0 and cleaned. Any missing information from the merged file was corrected by referring to the source documents. In the analysis, the term ‘non-prescription medicine use’ was restricted to antimicrobial agents (Amoxicillin, Ampicillin, Coartem, Cotrimoxazole, and Azithromycin) that were reportedly given to the children. Coartem is an anti-malarial agent containing Artemether and Lumefantrine, while Cotrimoxazole is antibacterial agent containing Sulfamethoxazole and Trimethoprim. All the other medicines reportedly given to the children during the recent illness were all over-the-counter agents.

Medicines that are obtained from the hospital were not considered as non-prescription agents. However, some individuals do not complete taking all the prescribed dose of medicines obtained from the hospital and decide to keep the remainder in the household. It is the use of such medicines stored at homes without further consulting a healthcare professional that was considered as ‘non-prescription use’ in this study.

According to the Uganda drug Act of 1972, all antimicrobial agents are categorized as prescription only medicines. All the other medicines (diphenhydramine, vitamin A, paracetamol, herbs, cold cap, and cough linctus reportedly given to the children in this study can be legally accessed over-the-counter in Uganda.

In Uganda, a drug shop is a private drug outlet which primarily should dispense only over-the-counter medicines while a pharmacy is usually in both public and private sector and dispense both over-the-counter and prescription only medicines. A pharmacy is supervised by holder of a degree in pharmacy while drug shops are supervised by Allied health professionals (holders of a diploma in medical training).

Descriptive statistics was generated for continuous variables using median and 75% interquartile range (IQR). Poisson regression was performed to assess the correlation of independent variables (age, sex, education level) of the childcare taker with the main outcome measure (non-prescription use of antimicrobial agents in management of symptoms of acute respiratory tract infection). Each variable was individually regressed with the main outcome measure and crude estimates determined. Multivariable regression model was then built to determine predictors of the use of self-medication in management of symptoms of upper respiratory tract infections among childcare takers. The variables to be included in the multivariable model were selected at 20% (0.2) level of significance. The final analysis in the multivariable model was performed at 95% confidence level.

## Results

### Demographic characteristics of children in households in Nakawa division, Kampala city

In this study, data on the use of medicines in children was collected from 390 child care takers. The median age of the children in each household was 3 (75% IQR: 1.8-5) years. Each household had 2-to-4 children and the majority were females, 52.8% (206/390) (Table 1). The majority, 44.8% (175/390) of the care takers of children interviewed had completed secondary level of education, while 6.2% (24/390) had never gone to school.

**Table 1:** Baseline characteristics of the children

Characteristic	Description	Children, *n* = 390	Caretakers, *n* = 390
Age	Median (IQR)	3 (1.8-5)	27 (24–32)
Sex	Female	206 (52.8%)	358 (91.8%)
	Male	184 (47.2%)	32 (8.2%)
Number of children in a household	Median (IQR)	2 (2–4)	–

*IQR* Interquartile Range, *n* sample size, % Percentage

### Prevalence of using non-prescription medicines to manage disease symptoms in children

The prevalence of non-prescription use of antimicrobial agents in managing symptoms of acute upper respiratory tract infections in children (≤12 years) was 44.8% (38.3, 52.2). The disease symptoms that majority of children reportedly had included flu, 84.9% (331/390), cough, 83.1% (324/390), undefined fever, 69.7% (272/390), lack of appetite, 44.1% (172/390) and general body weakness, 33.8% (132/390) (Table 2). When asked what disease the childcare takers thought the children were having, over half, 52.6% (203/390) said that it was malaria.

**Table 2:** Reported disease conditions of the children and how they were managed (*N* = 390)

Question	Description of answer	Proportion, *N* = 390 (%)
What symptom of ill health did the child have?	Running nose	370 (94.9%)
	Cough	324 (83.1)
	Difficulty breathing	91 (23.3%)
	Fever	272 (69.7%)
	Fast breathing	29 (7.4%)
	Lack of appetite	172 (44.1%)
	I do not remember	57 (14.6%)
What disease did you think the child was suffering from?	Malaria	205 (52.6%)
	Flu	261 (66.9%)
	Diarrhea	28 (7.2%)
	Pneumonia	20 (5.1%)
	Measles	21 (5.4%)
	I do not know	32 (8.2%)
Did the child receive any treatment?	Yes	368 (94.4%)
	No	22 (5.6%)
Who recommended the treatment	Childcare taker	156 (40%)
	Healthcare professional	203 (52.1%)
	Neighbor	5 (1.3%)
	Friends/relatives	4 (1.0%)
	Did not give any treatment	22 (5.6%)
Which medicine (s) did the child take?	Cough linctus	81 (20.8%)
	Amoxicillin	74 (18.9%
	Coartem (A-L)	116 (29.7%)
	Paracetamol	207 (53.1%)
	Ampicillin	6 (1.5%)
	Cotrimoxazole	72 (18.5%)
	Azithromycin	1 (0.3%)
	Diphenhydramine	60 (15.4%)
	Vitamin A	7 (1.8%)
	Cold cap	49 (12.6%)
	Herbal syrup	19 (4.9%)
What was the source of the medicine given to the child?	Pharmacy	73 (18.7%)
	Drug shop	51 (13.1%)
	Clinic/health facility	223 (57.2%)
	Home medicines	8 (2.1%)
	Friends/relatives	13 (3.3%)

*A-L* Artemether-Lumefantrine, *Cold cap* A combination that contains, decongestants (e.g. oxymetazoline, Phenylephrine and Pseudoephedrine), acetaminophen and antihistamine (e.g. diphenhydramine)

### Practices in the treatment of symptoms of acute upper respiratory tract infections (URTIs) among children (≤12 years) in households in Nakawa division

Of the 390 households visited, all the children had experienced illness (flu, body general weakness, cough, undefined fever, and lack of appetite) in the last two weeks (14 days) prior to the interview. However, treatment was reportedly sought and medicines given to only 368 (94.4%) of the children. The most common antimicrobial medicines given to the children for URTIs included, amoxicillin 18.9% (74/390), Cotrimoxazole 18.5% (72/390), ampicillin 1.5% (6/390) and azithromycin 0.3% (1/390) and Coartem 29.7% (116/390). The majority of the medicines given to the children were obtained from the hospital/clinic 57.26% (223/390) (Table 2).

Of the children who received medicines during the recent episode of acute upper respiratory tract infection, majority, 73.1% (269/368) completed taking the entire dose of medicines obtained for the illness (Table 3). Of the 85 children who did not complete taking all the entire dose, 79.1% (68/85) reported symptom improvement as the main reason. For the children whose symptoms did not resolve; 5.9% (22/368) were taken to the hospital/clinic, and in 2.7% (10/368) more medicines were given. Of the antimicrobial agents that was being given to the children, the dose of antimalarial agent (Coartem) that was obtained was all completed. While, 10.6% (9/85), 15.3% (13/85) did not complete taking the dose of antibacterial during the recent episode of acute respiratory tract infection (Table 3). The majority, 81% (298/368) of the children took more than one medicine at a time during the recent illness (Table 4). Most, 41.3% of the children who had symptoms of viral infections were given antimalarial agents by their childcare takers.

**Table 3:** Proportion of children who completed taking all the dose of medicine(s)

Drugs prescribed	Drug completion status			
	Yes	No	Still taking	Total
	N (%)	N (%)	N (%)	N (%)
Antimalarial	52 (14.1)	0 (0.0)	1 (0.3)	53 (14.4)
Antibacterial	20 (5.4)	9 (2.4)	0 (0.0)	29 (7.8)
NSAIDs	83 (22.6)	26 (7.1)	5 (1.4)	114 (30.9)
Antihistamine	29 (7.9)	15 (4.0)	1 (0.3)	45 (12.2)
Herbs	67 (18.2)	14 (3.8)	3 (0.8)	84 (22.8)
Vitamins	3 (0.3)	3 (0.8)	1 (0.3)	7 (1.9)
Others	28 (7.6)	5 (1.4)	1 (0.3)	34 (9.2)
Total	269 (73.1)	85 (23.1)	14 (3.8)	368 (100)

**Table 4:** Drug use practices in treatment of illnesses in children in households

Practice	Description	Proportion of children who received treatment, *N* = 368 (%)
Child completed the entire dose of medicine	Yes	269 (73.1)
	No	85 (23.1%)
	Still taking	14 (3.8)
Reason for not completing dose of medicine	Child improved	68 (17.4%)
	Child refused to take drug	1 (0.3%)
	Forgot to continue	1 (0.3%)
	Misplaced the drugs	15 (3.8%)
Gave the child more than one medicine at a time	Yes	298 (81%)
	No	67 (18.2%)
	I do not remember	3 (0.8%)

**Table 5:** Bivariate analysis of factors associated with antimicrobial self-medication

Factors	Crude PR	95%CI	*P*-value
Disease that the child was thought to be suffering from			
Malaria	1	–	–
Flu	1.36	0.98-1.88	0.06
Diarrhoea	1.56	0.99-2.46	0.05
Pneumonia	1.18	0.64-2.15	0.60
Others	1.11	0.70-1.74	0.67
Source of medication			
Pharmacy	1	–	–
Drug shop	1.40	1.09-1.79	0.009
Clinic	0.53	0.40-0.71	*p* < 0.001
Medicine container at home	1.78	1.45-2.18	*p* < 0.001
Others	1.23	0.81-1.87	0.32
Knowing risks of self-medication			
Yes	1	–	–
No	1.43	1.10-1.86	0.008

**Table 6:** Predictors of non-prescription antimicrobial use in children for URTIs

Predictors	Adjusted PR	95%CI	*P*-value
Source of medicines			
Pharmacy	1	–	–
Drug shop	1.45	1.14-1.85	0.002
Clinic	0.54	0.41-0.72	p < 0.001
Medicines found at home	1.80	1.39-2.33	p < 0.001
Others	1.28	0.83-1.98	0.27
Drugs given			
NSAID	1	–	–
Antimalarial	2.80	1.17-6.68	0.02
Antibacterial	1.35	0.88-2.08	0.17
Antihistamine	1.35	0.97-1.88	0.08
Others	1.23	0.85-1.80	0.28

*PR* Prevalence Ration, *NSAID* Non-Steroidal Anti-inflammatory Drug, *CI* Confidence Interval

In cases where the medicines obtained were not all used up, majority 70.6% (60/85) of the child care takers would keep the remaining medicine for use in case of future illness.

Some of the child caretakers, 12.8% (50/390) reported that they would recommend use of non-prescription medicines for other sick children in the neighborhood. The main reason for this recommendation was that when children are treated without consulting a healthcare professional, they improve, 52% (26/50). In addition, 25.3% (99/390) of the childcare takers reported that they would self-medicate the children under their care in future in case they fall ill. The majority, 76.4% (298/390) of children with symptoms of ill health were taking more than one kind of medicine at a time. As claimed by the child care takers, the disease symptoms improved in over a third of the children, 33.6% (131/390) after being given non-prescription medicines.

The majority, 74.6% (291/390) of childcare takers reported to be aware of the risks of taking drugs without a prescription from a healthcare professional. When asked to mention some of the medicines that should not be given to children, majority said they don’t know, 62.3% (243/390). However, others reported that cold cap 2.5% (10/390), Cotrimoxazole 1.8% (7/390), cough linctus 1.5% (6/390) and herbal syrup 0.8% (3/390) should not be given to children.

### Factors associated with non-prescription use of medicines in management of symptoms of acute upper respiratory tract infections in children (≤12 years) in Kampala city

At bivariate analysis, the child care takers who did not know the risks of non-prescription use of antimicrobial agents were, 1.43 (95%CI: 1.10-1.86) times more likely to give children antimicrobial agents without a prescription. While medicines obtained from the drug shop and leftover drugs at home were, 1.4 (1.09-1.79), and 1.78 (1.45-2.18) times more likely to be used without a prescription respectively (Table 5).

Based on multivariable analysis, medicines obtained from a drug shop and home were 45% and 80% more likely to be used without a prescription for managing illness in children. Antimalarial agents were over twice more likely to be given to children without a prescription. While medicines obtained from a clinic were 54% less likely to be given to children without a prescription (Table 6).

## Discussion

Most children get exposed to medicines early in life in many parts of the world. A study by Rossignali et al.*,* [15] showed that by age of five (5) years, over 70% of children in developed economies would have had at least one exposure to an antibacterial agent. A similarly high rate, over 80% exposure to antibacterial agents among children was also reported in developing countries [16]. In the current study, 94.4% of children (≤12 years) were given medicines in their most recent illness episode with the majority receiving antibacterial and antimalarial agents. This is higher than what was reported by a similar study in an urban setting in Brazil [17]. The variation in the extent of medicine use in children could be attributed to the differences in study methods such as recall period, age of children and the healthcare system. In a Brazilian study, children of age < 2 years were studied while in our study we included children of up to twelve years.

In the current study, over 90% of the children who had an episode of acute URTIs were given medicines. In addition, 44.8% of these children were given antimicrobial agents without a prescription. This is similar to the findings of a study done in Brazil [4]. The high exposure to medicines in children found in the current study coupled with their non-prescription use potentially increase the risk of adverse drug reactions. In addition, non-prescription use of medicines is commonly associated with inappropriate use and could result in masking symptoms of more severe illness, consumption of expired drugs and or development of drug resistance [18]. The risk of adverse effects is of particular interest especially in children. This is because drug metabolizing enzyme systems are not yet fully developed and thus disproportionately exposing children to toxic drug effects due to bio-accumulation [19, 20]. Child caretakers thus need to be educated on the dangers of giving medicines to children. In developing countries, easy accessibility of medicines in the private sector coupled with the inadequate public sector healthcare delivery potentially influence the high prevalence of self-medication [21, 22]. In Uganda, cost sharing in public healthcare sector was abolished over a decade ago. However, it’s common for medicines and other medical supplies to be out of stock in public health facilities. As such the population resorts to the private sector to seek healthcare services.

The majority of child caretakers reported to have knowledge of the medicines that they were giving to the children. However, in our study when the childcare takers were asked which medicines should not be given to children over half of the respondents did not know. A finding similar to that of a previous study by Huang et al.*,* [23] that also reported misconceptions on the knowledge of appropriate indications and efficacy of antibiotics among child caretakers. This is of concern especially as some of the childcare takers gave children medicines without consulting any medical professional. In addition, most children were given multiple medications at a time further increasing the risk of drug interactions, adverse reactions and misuse. Our study also found that a majority of children were being given antibacterial and antimalarial agents for flu, cough, and undefined fever. A finding similar to that of a previous study by Grigoryan et al.*,* [24] which reported use of antibacterial agents for treatment of viral infections such as flu. A study on non-prescription use of antibiotics for children in an urban community in Mongolia found that most people had misconceptions about antibiotic use in flu, cough, and sore throat [25].

In this study most childcare takers reported disease symptom resolution among children managed using non-prescription medicines. This perceived positive outcome could potentially have a bearing on medicine use practices in communities. As reported in our study, majority of the childcare takers would recommend other members in the neighborhood to give their children antimicrobial agents without a prescription. In addition, most childcare takers would self-medicate the children under their care in case of future illness. There is generally limited information on the safety of medicines in children [7, 8]. In addition, majority of the child care takers who gave medicines to the children had limited education. This is likely to further increase the risk of inappropriate treatment and resistance development [20].

The study had some limitations, since we used self-reported practices of childcare takers on how they managed episodes of acute URTIs in the children under their care, it is possible that there was bias in the recall of the medicines used. However this could have been minimized in the current study by the short recall period (two weeks or 14 days) used during data collection.

## Conclusions

Nearly all the children who had symptoms of acute URTIs in the two week period prior to our field data collection were administered medicines. In addition, over a third of the children were given antimicrobial (antibacterial and antimalarial) agents without medical advice by a healthcare professional. There is need to educate the public on the dangers of use of antimicrobial agents without seeking professional advice and proper diagnosis of the cause of illness in children. In addition, regulation of over-the-counter dispensing of antimicrobial agents especially in private drug outlets needs to be enforced in the country.
